# Integrated transcriptomic, protein, and MicroRNA profiling reveals a conserved pyroptosis-related molecular signature across breast cancer subtypes

**DOI:** 10.3389/fmolb.2026.1759944

**Published:** 2026-03-04

**Authors:** Agata Panfil, Tomasz Sirek, Agata Sirek, Nikola Zmarzły, Michalina Wróbel, Zbigniew Wróbel, Kacper Boroń, Dariusz Boroń, Piotr Ossowski, Martyna Stefaniak, Paweł Ordon, Grzegorz Wyrobiec, Piotr Wyrobiec, Beniamin Oskar Grabarek

**Affiliations:** 1 Collegium Medicum, WSB University, Dabrowa Gornicza, Poland; 2 Department of Plastic Surgery, Faculty of Medicine, Academia of Silesia, Katowice, Poland; 3 Department of Plastic and Reconstructive Surgery, Hospital for Minimally Invasive and Reconstructive Surgery in Bielsko-Biała, Bielsko-Biala, Poland; 4 Medi-Lab SC, Wróbel i wspólnicy, Świdnica, Poland; 5 Faculty of Medicine and Health Sciences, Andrzej Frycz Modrzewski University in Kraków, Kraków, Poland; 6 Department of Gynecology and Obstetrics, TOMMED Specjalisci od Zdrowia, Katowice, Poland; 7 Department of Gynecology and Obstetrics with Gynecologic Oncology, Ludwik Rydygier Memorial Specialized Hospital, Kraków, Poland; 8 Department of Histology and Cell Pathology in Zabrze, Faculty of Medical Sciences in Zabrze, Medical University of Silesia in Katowice, Katowice, Poland

**Keywords:** breast cancer, cell death, micro RNA, molecular marker, pyroptosis

## Abstract

**Background:**

Pyroptosis, an inflammatory form of programmed cell death, has been implicated in tumor progression, yet its molecular contribution across breast cancer subtypes remains poorly defined.

**Methods:**

To characterize pyroptosis-related alterations, we analyzed tumor and matched control tissues from five molecular subtypes of breast cancer using genome-wide messenger RNA and microRNA microarrays, quantitative polymerase chain reaction, enzyme-linked immunosorbent assays, and protein–protein interaction analysis. We identified predicted microRNA–messenger RNA regulatory relationships and constructed a pyroptosis index and inflammasome activation score. To contextualize these findings, temporal expression changes were evaluated in a cryoablation model of benign fibroadenoma.

**Results:**

Nine genes associated with inflammatory and apoptotic signaling—*CXCL8*, *BCL2*, *BAX*, *CASP1*, *CASP9*, *TP53*, *CDKN1A*, *CDKN1B*, and *MMP9*—consistently distinguished cancerous from control tissue across all subtypes at both messenger RNA and protein levels. Aggressive subtypes, particularly human epidermal growth factor receptor 2–enriched and triple-negative tumors, exhibited pronounced activation of inflammasome-related pathways, elevated pyroptosis index and inflammasome activation score values, and coordinated suppression of cell-cycle inhibitors. Predicted microRNA regulators, including microRNA 140-3p, microRNA 124-3p, microRNA 300, microRNA 30a-3p, microRNA 30d-3p, and microRNA 608, showed patterns consistent with loss of post-transcriptional restraint in high-grade tumors. In fibroadenoma, pyroptosis-associated expression changes were rapid and transient, whereas malignant tissue displayed a consistent, subtype-dependent elevation of pyroptosis-related markers at the time of resection.

**Conclusion:**

This integrative analysis identifies a conserved pyroptosis-related molecular signature that deepens understanding of inflammatory programmed cell death in breast cancer and highlights interconnected pathways with diagnostic, prognostic, and therapeutic relevance.

## 1 Introduction

Pyroptosis is an inflammatory form of programmed cell death characterized by membrane pore formation, cellular swelling, and rapid release of pro-inflammatory cytokines (Yu et al., 2021). Pyroptosis derives from the terms “pyro,” meaning fire and reflecting its strongly inflammatory nature, and “ptosis,” meaning falling, a suffix shared with other forms of programmed cell death (D’Souza and Heitman, 2001). Although pyroptosis is distinct from apoptosis, the two processes share certain morphological features, including DNA fragmentation and chromatin condensation (Ban et al., 2024). Notably, cells undergoing pyroptosis exhibit marked swelling, and numerous bubble-like protrusions form on the plasma membrane prior to its final rupture (Chen et al., 2016). This process is mainly driven by the activation of intracellular protein complexes known as inflammasomes, which stimulate caspase-1 (cysteine-aspartic protease 1; CASP1) and, in the non-canonical pathway, caspase-4 and caspase-5 (CASP4 and CASP5). The non-canonical pyroptosis pathway is activated primarily in response to intracellular lipopolysaccharide and is mediated by CASP4 and CASP5 in humans, leading to gasdermin cleavage and secondary inflammasome activation. Unlike the canonical pathway, which depends on pattern-recognition receptor–driven inflammasome assembly and caspase-1 activation, the non-canonical pathway directly links innate immune sensing to inflammatory cell death and can amplify tumor-associated inflammation independently of classical inflammasome sensors (Ghait et al., 2023; Yang et al., 2015). Activated caspases cleave gasdermin family members—particularly gasdermin D (GSDMD)—leading to pore formation and release of interleukin-1β (IL-1β) and interleukin-18 (IL-18) (Zhang and Wirtz, 2022). Although pyroptosis is a natural component of host defense, increasing evidence shows that its dysregulation contributes to tumor development, including breast cancer (Zhou et al., 2024; Tan et al., 2021).

In breast cancer, pyroptosis-related signaling plays complex and sometimes opposing roles (Chen et al., 2022; Chen et al., 2023; You et al., 2023). On one hand, activation of pyroptosis can suppress tumor growth by inducing cancer cell death (Chen et al., 2022; Chen et al., 2023; You et al., 2023). On the other hand, chronic inflammasome activity and continuous cytokine release may support a pro-tumor inflammatory microenvironment, promote immune evasion, and facilitate metastasis (Chen et al., 2022; Chen et al., 2023; You et al., 2023). The balance between these effects is influenced by the expression of genes encoding key pyroptosis-associated proteins, such as NOD-like receptor family pyrin domain containing 3 (NLRP3), CASP1, CASP4/5, IL-1β, IL-18, and GSDMD (Huang et al., 2025).

MicroRNAs (miRNAs), small non-coding RNA molecules that regulate gene expression at the post-transcriptional level (O’Brien et al., 2018), are increasingly recognized as critical modulators of pyroptosis. By binding to messenger RNA (mRNA) targets, miRNAs fine-tune inflammasome activity, cytokine production, and gasdermin signaling. Alterations in miRNA expression may therefore shift the balance between tumor-suppressive and tumor-promoting pyroptotic pathways, influencing disease progression, clinical phenotype, and treatment response (Lee et al., 2015; Hu et al., 2021).

Despite growing interest in the interplay between pyroptosis and miRNA-mediated regulation, comprehensive studies examining these interactions in breast cancer remain limited. The aim of this study is to analyze changes in the expression of genes encoding key pyroptosis-related proteins and the miRNAs that regulate them, providing new insights into the molecular mechanisms linking inflammatory cell death with breast cancer biology.

## 2 Materials and methods

### 2.1 Study design

This research builds upon our previous studies, extending the analysis to additional molecular pathways relevant to pyroptosis in breast cancer (Sirek et al., 2024a; Sirek et al., 2024b; Sirek et al., 2024c; Sirek et al., 2024a).

The study was designed to comprehensively evaluate pyroptosis-related molecular alterations in breast cancer across five molecular subtypes. Tumor tissue and corresponding control samples were obtained from patients diagnosed with luminal A, luminal B HER2-negative, luminal B HER2-positive, non-luminal HER2-positive, and triple-negative breast cancer (TNBC). For all collected tissue specimens, a multilevel molecular analysis was performed, including genome-wide mRNA and microRNA (miRNA) expression profiling using microarray technology. Selected differentially expressed genes were subsequently validated by quantitative reverse transcription polymerase chain reaction (qRT-PCR), while corresponding protein levels were assessed with enzyme-linked immunosorbent assay (ELISA).

In the second part of the study, an additional comparative group was included, consisting of patients diagnosed with breast fibroadenoma who were qualified for cryoablation. Peripheral blood samples were collected from these patients twice: immediately before the procedure and again at 7 days post-cryoablation. Gene expression in blood samples was quantified using qRT-PCR, and circulating protein levels were measured by ELISA.

The analyses performed in the fibroadenoma group focused exclusively on those genes and encoded proteins that, in the breast cancer microarray experiments, demonstrated consistent differential expression between tumor and control tissues regardless of cancer subtype. This allowed for the evaluation of whether early molecular responses observed in circulating blood following cryoablation reflect key pyroptosis-related pathways identified in breast cancer tissue.

### 2.2 Study population

#### 2.2.1 Patients with breast cancers

The study included patients representing five molecular subtypes of breast cancer: luminal A (n = 130), luminal B HER2– (n = 100), luminal B HER2+ (n = 96), non-luminal HER2+ (n = 36), and triple-negative breast cancer (TNBC; n = 43). During surgical treatment, tumor tissue and a margin of histologically normal tissue were excised; the latter served as the control.

All cases were staged as T1N0M0 according to the Tumor–Node–Metastasis (TNM) classification (O’Sullivanet al., 2017).

Intraoperative consultation with the pathology team confirmed the adequacy of tumor-free margins using immunohistochemistry. If necessary, resection was extended. Based on these assessments, paired samples of tumor-infiltrated tissue and adjacent unaffected tissue were collected for molecular analyses.

The research included patients representing five molecular subtypes of breast cancer: luminal A (n = 130), luminal B HER2– (n = 100), luminal B HER2+ (n = 96), non-luminal HER2+ (n = 36), and TNBC (n = 43). During surgical treatment, both tumor tissue and a margin of histologically normal tissue were excised; the latter served as the control.

All cases were staged as T1N0M0 according to the Tumor–Node–Metastasis (TNM) classification (O’Sullivanet al., 2017).

The Luminal A subtype included 23 cases (18%) with G1 histological malignancy, 48 cases (37%) with G2, and 59 cases (45%) with G3. In terms of age distribution, 43 patients (33%) were younger than 50 years, while 87 patients (67%) were older than 50 years. The mean BMI in this group was 30.78 ± 2.76 kg/m^2^.

The Luminal B HER2-negative subtype consisted of 31 cases (31%) classified as G1, 57 cases (57%) as G2, and 12 cases (12%) as G3. Among these patients, 32 (32%) were below 50 years of age and 68 (68%) were above 50 years. The mean BMI for this group was 30.18 ± 4.56 kg/m^2^.

For the Luminal B HER2-positive subtype, 23 cases (24%) were graded as G1, 57 cases (59%) as G2, and 16 cases (17%) as G3. A total of 19 patients (20%) were younger than 50 years, whereas 77 (80%) were older than 50 years. The mean BMI was 32.09 ± 6.19 kg/m^2^.

The non-luminal HER2-positive subtype comprised 9 cases (25%) with G1, 12 cases (33%) with G2, and 15 cases (42%) with G3 malignancy. In this group, 9 patients (25%) were below 50 years of age and 27 (75%) were above 50 years. The mean BMI was 33.18 ± 5.67 kg/m^2^.

The TNBC subtype included 14 cases (32%) with G1, 21 cases (49%) with G2, and 8 cases (19%) with G3. A total of 10 patients (23%) were younger than 50 years and 33 (77%) were older than 50 years. The mean BMI in this subtype was 34.67 ± 2.98 kg/m^2^.

Intraoperative consultation with the pathology team was used to confirm whether tumor removal included an adequate margin of uninvolved tissue (verified via immunohistochemistry). If the margin was insufficient, the surgeon extended the resection. Based on these assessments, paired samples of tumor-infiltrated tissue (study group) and adjacent unaffected tissue (control group) were collected for molecular analyses.

#### 2.2.2 Patients with fibroadenoma

A total of 34 patients with diagnosed breast fibroadenoma were included in the study and qualified for cryoablation using the IceCure ProSense™ (IceCure Medical HQ, Caesarea, Israel) system according to the manufacture’s protocol. The mean age of the participants was 35.87 ± 4.11 years, and the mean BMI was 26.15. ± 4.98 kg/m^2^. For each patient, peripheral blood samples were collected at the following time points: before the procedure (T0), 30–60 min after cryoablation (T1), 8–12 h post-procedure (T2), 48–72 h (T3), 7 days (T4), 1 month (T5), and 3 months (T6).

### 2.3 Extraction of total RNA

Total RNA was isolated from tissue samples using TRIzol reagent (Invitrogen, Carlsbad, CA, United States; Cat. No. 15596026) following the manufacturer’s protocol. Commercially available PAXgene Blood RNA kit (Qiagen, Valencia, CA, United States, Cat No. 762174) was used to extract the total RNA from the whole blood as per the manufacturer’s protocol. Purification of RNA was subsequently performed with the RNeasy Mini Kit (QIAGEN, Hilden, Germany; Cat. No. 74104). To eliminate residual genomic DNA, samples were treated with DNase I (Fermentas International Inc., Burlington, ON, Canada; Cat. No. 18047019).

RNA integrity was verified using 1% agarose gel electrophoresis stained with 0.5 mg/mL ethidium bromide, enabling visualization of rRNA bands. RNA concentration and purity were quantified spectrophotometrically by measuring absorbance at 260 nm.

### 2.4 Microarray profiling of pyroptosis related genes

Genes associated with pyroptosis were identified using the GeneCards human gene database (accessed 10 November 2025) by searching the keyword “pyroptosis.” This search yielded 877 mRNAs that met the inclusion criteria.

Expression analyses of these genes in tumor versus control samples were performed using the HG-U133_A2 microarray platform (Affymetrix, Santa Clara, CA, United States) together with the GeneChip™ 3′IVT PLUS Reagent Kit (Affymetrix; Cat. No. 902416). The analytical workflow followed previously described and manufacturer-recommended procedures. Among the 22,277 probes present on the microarray, 65 were specific to the pyroptosis.

The protocol included synthesis of double-stranded complementary DNA (cDNA), *in vitro* transcription to amplify RNA (aRNA), fragmentation, and hybridization to the arrays. Fluorescence detection was performed using the Affymetrix GeneArray Scanner 3000 7G, with data acquisition via GeneChip® Command Console® Software. This approach enabled a detailed assessment of expression alterations across pyroptosis-associated transcripts.

### 2.5 Global profiling of pyroptosis-related miRNAs and their predicted targets

To explore miRNAs potentially regulating pyroptosis-associated gene expression, samples were analyzed using the GeneChip miRNA 2.0 Array (Affymetrix). All procedures adhered strictly to the manufacturer’s instructions to ensure reproducibility and analytical reliability.

Differentially expressed miRNAs were identified by comparing tumor and control tissues. Potential miRNA–mRNA regulatory interactions were predicted using TargetScan (http://www.targetscan.org/) (Agarwal et al., 2015) and miRanda/mirDB (http://mirdb.org) (Chen and Wang, 2020). These databases provide computational models predicting miRNA binding based on sequence complementarity and evolutionary conservation (Chen and Wang, 2020; Liu and Wang, 2019).

For the purposes of this study, targets with prediction scores >80 were considered highly reliable. Predictions <60 were treated with caution and interpreted only in conjunction with additional evidence. Integrating data from multiple prediction tools strengthened confidence in inferred miRNA–mRNA interactions and helped delineate regulatory networks associated with pyroptosis-related signaling.

### 2.6 Quantitative reverse transcription PCR (qRT-PCR) validation

Selected genes identified through microarray analysis were validated using quantitative reverse transcription polymerase chain reaction (qRT-PCR). Experiments were conducted with the SensiFast SYBR No-ROX One-Step Kit (Bioline, London, UK), following the manufacturer’s protocol.

Gene expression levels were calculated using the 2^−ΔΔCT^ method, where a fold change of 1 represented the control group, values >1 indicated upregulation, and values <1 indicated downregulation. β-actin (ACTB) served as the endogenous control. Primer sequences used for amplification are provided in Table 1.

**TABLE 1:** Primer sequences used in the experiment.

mRNA	Nucleotide sequence (5′-3′)
*CXCL8*	Forward: CAGCCAACAGGTGAGAATGA Reverse: 5′TTGAAGGATGTTCCCAGAGG
*BCL2*	Forward: GATTGTGGCCTTCTTTGAG Reverse: GTTCCACAAAGGCATCC
*BAX*	Forward: CCTGTGCACCAAGGTGCCGGAACT Reverse: CCACCCTGGTCTTGGATCCAGCCC
*CASP1*	Forward: CAACTACAGAAGAGTTTGAGG Reverse: AACATTATCTGGTGTGGAAG
*CASP9*	Forward: CTCTACTTTCCCAGGTTTTG Reverse: TTTCACCGAAACAGCATTAG
*TP53*	Forward: ACCTATGGAAACTACTTCCTG Reverse: ACCATTGTTCAATATCGTCC
*CDKN1A*	Forward: CAGCATGACAGATTTCTACC Reverse: CAGGGTATGTACATGAGGAG
*CDKN1B*	Forward: AACCGACGATTCTTCTACTC Reverse: TGTTTACGTTTGACGTCTTC
*MMP9*	Forward: GAGTTCCCGGAGTGAGTTGA Reverse: AAAGGTGAGAAGAGAGGGCC
*ACTB*	Forward: TCACCCACACTGTGCCCATCTACGA Reverse: CAGCGGAACCGCTCATTGCCAATGG

*CXCL8*, C-X-C Motif Chemokine Ligand 8 (also known as Interleukin-8, IL-8); *BCL2*, B-Cell Lymphoma 2, *BAX*, BCL2-Associated X Protein; *CASP1,* Caspase-1; *CASP9*, Caspase-9; *TP53*, Tumor Protein p53; *CDKN1A*, Cyclin-Dependent Kinase Inhibitor 1A (p21); *CDKN1B*, Cyclin-Dependent Kinase Inhibitor 1B (p27); *MMP9*, Matrix Metallopeptidase 9; *ACTB*, β-actin.

### 2.7 Calculation of the pyroptosis index (PI)

To quantify the overall activation of pyroptosis-related signaling across breast cancer molecular subtypes, a Pyroptosis Index (PI) was constructed based on the expression profiles of nine core pyroptosis-associated genes that were consistently and significantly dysregulated in tumor tissue compared with matched controls, irrespective of subtype. These genes were identified from the microarray dataset as the most robust subtype-independent markers and included: *CXCL8, BAX, CASP1, CASP9, TP53, MMP9, BCL2, CDKN1A,* and *CDKN1B*.

Based on their established biological functions, genes were categorized into two functional groups reflecting opposing roles in the regulation of pyroptotic and inflammatory cell-death pathways. The pro-pyroptotic group comprised *CXCL8*, *BAX*, *CASP1*, *CASP9*, *TP53*, and *MMP9*, which promote inflammasome activation, caspase signaling, and inflammatory cell death. The anti-pyroptotic (pyroptosis-suppressive) group included *BCL2*, *CDKN1A*, and *CDKN1B*, genes involved in inhibition of programmed cell death and maintenance of cell-cycle control.

To account for inter-gene variability in expression magnitude, fold-change values were first log_2_-transformed using a log_2_ (1 + FC) transformation to stabilize variance and reduce the influence of extreme values. Subsequently, for each gene, transformed expression values were standardized across the study cohort using Z-score normalization (mean = 0, standard deviation = 1). These standardized values were then used for index construction.

For each sample, the PI was calculated as the difference between the mean Z-score of pro-pyroptotic genes and the mean Z-score of anti-pyroptotic genes, according to the formula:

$$\text{PI}=\text{mean}\left(Z_{\text{pro}‐\text{pyroptotic}\;\text{genes}}\right)-\text{mean}\left(Z_{\text{anti}‐\text{pyroptotic}\;\text{genes}}\right)$$

Higher PI values indicate stronger activation of pyroptosis-associated and inflammatory cell-death pathways, whereas lower or negative values reflect reduced pyroptotic signaling and relative dominance of survival or cell-cycle inhibitory mechanisms.

### 2.8 Calculation of the inflammasome activation score (IAS)

The degree of inflammasome pathway activation was quantified using an Inflammasome Activation Score (IAS), calculated exclusively from experimentally measured microarray expression data. The IAS was constructed based on a curated panel of ten inflammasome-associated genes that were directly represented on the microarray platform and passed quality-control criteria. These genes reflect key functional components of inflammasome signaling and included canonical sensors, adaptor proteins, inflammasome-regulated cytokines, signaling regulators, and downstream effector caspases: *IL1B*, *IL18*, *NLRP3*, *PYCARD* (*ASC*), *TLR9*, *RIPK1*, *TNF*, *STING1*, *JAK3*, and *CASP1*.

For each gene, log_2_ fold-change (log_2_FC) values were calculated relative to matched control tissue using the same preprocessing and normalization pipeline applied to all transcriptomic analyses in this study.

For each sample, the IAS was calculated as the arithmetic mean of the log_2_ fold-change values of the ten inflammasome-related genes, according to the formula:

$$\text{IAS}=\text{mean}\left(\log_{2}{\text{FC}}_{\text{inflammasome}\;\text{genes}}\right)$$

Higher IAS values indicate stronger activation of inflammasome signaling, increased IL-1β and IL-18–associated inflammatory activity, and an overall intensified pro-inflammatory tumor microenvironment.

### 2.9 Protein quantification by enzyme-linked immunosorbent assay (ELISA)

Venous blood samples were obtained from all participants by standard antecubital venipuncture using sterile, single-use equipment. Approximately 5 mL of peripheral blood was collected into serum-separating tubes (SST) containing a clot activator. The samples were allowed to clot undisturbed for 30–45 min at room temperature. Subsequently, the tubes were centrifuged at 1,500–2,000 × g for 10–15 min to separate the serum fraction. The resulting serum was carefully aliquoted into sterile microtubes to avoid repeated freeze–thaw cycles. All samples were stored at −80 °C until analysis. Quantification of target proteins was performed using commercially available ELISA kits according to the manufacturer’s protocol.

Protein concentrations of the selected targets were measured using commercially available ELISA kits, following the manufacturer’s instructions for each assay. The following kits (all from MyBioSource, Inc., San Diego, CA, United States) were employed: Human Interleukin-8 (IL-8) ELISA Kit (MBS703104); Human BCL-2–Associated X Protein (BAX) ELISA Kit (MBS701787); Human Caspase-1/ICE ELISA Kit (MBS163158); Human Caspase-9 ELISA Kit (MBS704900); Human P53 ELISA Kit (MBS041908); Human Antioncogene p21/CDKN1A ELISA Kit (MBS731903); and Human Cyclin-Dependent Kinase Inhibitor 1B (CDKN1B) ELISA Kit (MBS2122089); Human Matrix Metalloproteinease-9 (92 kDa Type IV Collagenase) ELISA Kit (MBS8415331).

### 2.10 Statistical procedures

Statistical analyses were performed using Statistica 13.0 PL (StatSoft, Kraków, Poland) and Transcriptome Analysis Console (Affymetrix). Normality of distribution was checked using the Shapiro–Wilk test (p < 0.05). Depending on data characteristics, group comparisons were conducted using analysis of variance (ANOVA) with Benjamini–Hochberg correction and Tukey’s *post hoc* test, or the Student’s t-test.

Overall survival analyses were performed with the Kaplan–Meier Plotter (http://kmplot.com/) (Győrffy, 2024a; Győrffy, 2024b). Gene–gene and protein–protein association networks were examined using the STRING database (version 11.0; accessed 20 November 2025). STRING network enrichment was evaluated by the Log10 (observed/expected) strength parameter and the false discovery rate (FDR), adjusted using the Benjamini–Hochberg method (Szklarczyk et al., 2023).

### 2.11 Determination of sample size

Sample size estimation was performed using an online sample-size calculator (Kalkulator doboru próby, 2022), assuming a 95% confidence interval and considering that approximately 19,620 women were diagnosed with breast cancer in Poland in 2019 (Krajowy Rejestr Nowotworów, 2019), the calculated minimum sample size required was 377 participants.

For contextual comparison, the distribution of breast cancer subtypes based on national epidemiological data (Dai et al., 2015; Jassem et al., 2020) was contrasted with their representation in our study population. Reported proportions in the literature include luminal A (23.7%), luminal B HER2– (38.8%), luminal B HER2+ (14%), HER2+ (11.2%), and TNBC (12.3%). These values correspond closely to the subtype distribution observed in our cohort.

## 3 Results

### 3.1 Gene expression profile assessed with mRNA microarrays

Out of the 877 mRNAs associated with pyroptosis, ANOVA identified 48 transcripts that significantly differentiated tumor tissue from control samples (p < 0.05, log_2_FC >3 or <−3). Subsequent Tukey’s *post hoc* test revealed that 9 mRNAs consistently distinguished neoplastic tissue from non-tumor controls regardless of the breast cancer subtype, indicating their subtype-independent discriminatory potential.

**FIGURE 1:**
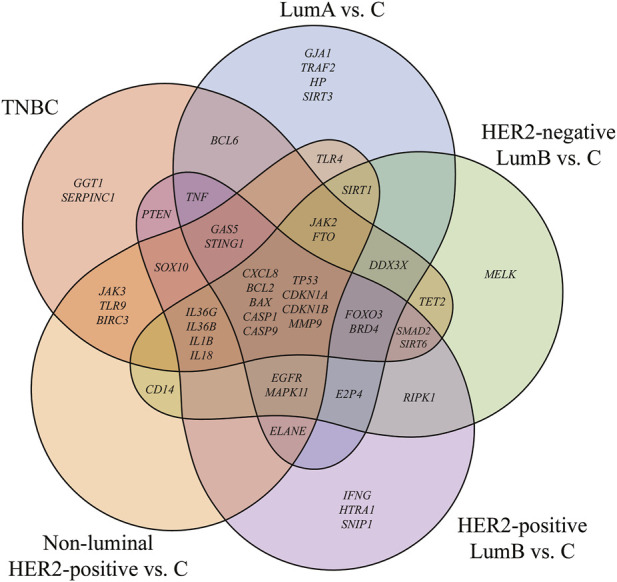
Venn diagram of genes differentiating breast cancer from the control. LumA, luminal A; LumB, luminal B; HER2, human epidermal growth factor receptor 2; TNBC, triple-negative breast cancer; C, control; *CXCL8*, C-X-C Motif Chemokine Ligand 8 (also known as Interleukin-8, IL-8); *BCL2*, B-Cell Lymphoma 2; *BAX*, BCL2-Associated X Protein; *CASP1,* Caspase-1; *CASP9*, Caspase-9; *TP53*, Tumor Protein p53; *CDKN1A*, Cyclin-Dependent Kinase Inhibitor 1A (p21); *CDKN1B*, Cyclin-Dependent Kinase Inhibitor 1B (p27); *MMP9*, Matrix Metallopeptidase 9; *GJA1*, Gap Junction Protein Alpha 1 (Connexin 43); *BCL6*, B-Cell Lymphoma 6; *TRAF2*, TNF Receptor–Associated Factor 2; *HP*, Haptoglobin; *SIRT3*, Sirtuin 3; *MELK*, Maternal Embryonic Leucine Zipper Kinase; *RIPK1*, Receptor-Interacting Serine/Threonine-Protein Kinase 1; *IFNG*, Interferon Gamma; *HTRA1*, HtrA Serine Peptidase 1; *SNIP1*, Smad Nuclear Interacting Protein 1; *IL36G*, Interleukin 36 Gamma; *IL36B*, Interleukin 36 Beta; *IL1B*, Interleukin 1 Beta; *IL18*, Interleukin 18; *JAK3*, Janus Kinase 3; *TLR9*, Toll-Like Receptor 9; *BIRC3*, Baculoviral IAP Repeat Containing 3; *CD14*, CD14 Molecule; *GGT1*, Gamma-Glutamyltransferase 1; *SERPINC1*, Serpin Family C Member 1 (Antithrombin III); *PTEN*, Phosphatase and Tensin Homolog; *SOX10*, SRY-Box Transcription Factor 10; *TNF*, Tumor Necrosis Factor; *GAS5*, Growth Arrest Specific 5; *STING1*, Stimulator of Interferon Response cGAMP Interactor 1; *EGFR*, Epidermal Growth Factor Receptor; *MAPK11*, Mitogen-Activated Protein Kinase 11 (p38β); *ELANE*, Elastase, Neutrophil Expressed; *JAK2*, Janus Kinase 2; *FTO*, Fat Mass and Obesity-Associated Protein; *DDX3X*, DEAD-Box Helicase 3 X-Linked; *TET2*, Ten-Eleven Translocation Methylcytosine Dioxygenase 2; *SMAD2*, SMAD Family Member 2; SIRT6, Sirtuin 6; *FOXO3*, Forkhead Box O3; *BRD4*, Bromodomain Containing 4; *E2F4*, E2F Transcription Factor 4; *SIRT1*, Sirtuin 1; *TLR4*, Toll-Like Receptor 4.

A graphical summary of these findings is presented in Figure 1, where a Venn diagram illustrates the overlap of differentially expressed mRNAs across the five breast cancer subtypes and highlights the subset of universally altered transcripts.

### 3.2 Microarray expression profiling of pyroptosis-related genes across breast cancer subtypes

Microarray analysis confirmed substantial differences in the expression of nine pyroptosis-associated genes across all breast cancer subtypes compared with control tissue (Table 2). *CXCL8* showed consistent and strong upregulation in all subtypes, with the highest expression in TNBC (8.18–8.21-fold) and non-luminal HER2+ tumors (7.21–7.65-fold). *BCL2* was markedly overexpressed in luminal tumors (5.19–5.41-fold in Luminal A; 3.19–3.43-fold in Luminal B HER2−; 4.01–4.17-fold in Luminal B HER2+) but demonstrated significant downregulation in non-luminal HER2+ (−3.19 to −4.19) and TNBC (−4.12 to −4.22). The pro-apoptotic gene *BAX* displayed progressive upregulation from luminal to highly aggressive subtypes, reaching the highest levels in TNBC (9.18–9.87-fold) and non-luminal HER2+ cancers (9.12–9.18-fold). *CASP1* and *CASP9* showed a similar pattern of stepwise activation, with modest increases in luminal tumors (3.1–4.7-fold) and markedly elevated expression in non-luminal HER2+ and TNBC, where *CASP1* reached 7.01–7.32-fold and *CASP9* 5.10–5.18-fold. *TP53* was moderately upregulated in luminal subtypes (3.11–3.41-fold) and strongly increased in HER2+ (5.91–6.43-fold) and TNBC (7.89–7.92-fold).

**TABLE 2:** Differential expression (log_2_ fold change) of pyroptosis-associated mRNAs across five breast cancer subtypes compared with control tissue.

Id	mRNA	Luminal A vs. control	Luminal B HER2− vs. control	Luminal B HER2+ vs. control	Non-luminal HER2+ vs. control	TNBC vs. control
202859_x_at	*CXCL8*	3.08	3.33	5.76	7.65	8.18
211506_s_at		3.08	3.29	5.81	7.21	8.21
203684_s_at	*BCL2*	5.23	3.19	4.01	−3.19	−4.12
203685_at		5.41	3.43	4.02	−3.21	−4.18
207004_at		5.19	3.28	4.17	−3.43	−4.22
207005_s_at		5.29	3.19	4.17	−4.19	−4.19
208478_s_at	*BAX*	3.18	4.55	5.98	9.12	9.87
211833_s_at		3.12	5.01	6.02	9.18	9.18
206011_at	*CASP1*	3.91	4.48	5.32	5.91	7.11
209970_x_at		3.54	4.56	5.21	5.89	7.19
211366_x_at		3.81	4.49	5.10	5.98	7.32
211367_s_at		3.44	4.61	5.61	5.89	7.01
211368_s_at		3.87	4.71	5.42	5.73	7.21
203984_s_at	*CASP9*	3.10	3.91	4.15	4.76	5.10
210775_x_at		3.21	3.87	4.19	4.81	5.18
240437_at		3.19	3.90	4.81	4.91	5.14
201746_at	*TP53*	3.11	3.41	5.91	6.19	7.89
211300_s_at		3.12	3.32	5.98	6.43	7.92
202284_s_at	*CDKN1A*	3.45	4.21	4.98	−4.18	−8.19
209112_at	*CDKN1B*	3.11	3.99	−3.14	−6.16	−17.55
203936_s_at	*MMP9*	3.19	3.87	4.56	9.19	12.91

HER2, human epidermal growth factor receptor 2; TNBC, triple-negative breast cancer; *CXCL8*, C-X-C Motif Chemokine Ligand 8 (also known as Interleukin-8, IL-8); *BCL2*, B-Cell Lymphoma 2, *BAX*, BCL2-Associated X Protein; *CASP1,* Caspase-1; *CASP9*, Caspase-9; *TP53*, Tumor Protein p53; *CDKN1A*, Cyclin-Dependent Kinase Inhibitor 1A (p21); *CDKN1B*, Cyclin-Dependent Kinase Inhibitor 1B (p27); *MMP9*, Matrix Metallopeptidase 9.

Cell-cycle regulators showed divergent patterns: *CDKN1A (p21)* was elevated in luminal tumors (3.45–4.98-fold) but suppressed in aggressive subtypes, particularly in TNBC (−8.19). *CDKN1B (p27)* followed a similar trend, remaining upregulated in luminal cancers (3.11–3.99-fold) and strongly downregulated in both HER2+ (−3.14 to −6.16) and TNBC (−17.55).

Finally, *MMP9* exhibited modest upregulation in luminal cancers (3.19–3.87-fold) but was dramatically increased in non-luminal HER2+ (9.19-fold) and especially TNBC (12.91-fold), consistent with its known role in invasiveness.

### 3.3 qRT-PCR validation of pyroptosis-related gene expression

**FIGURE 2:**
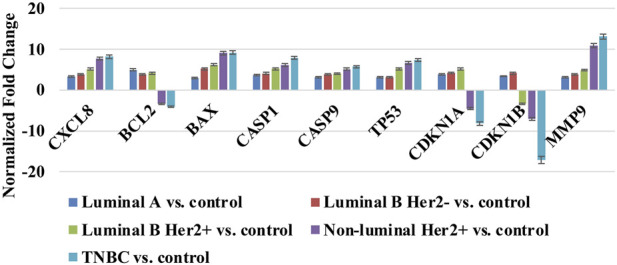
Validation of differential expression of nine pyroptosis-associated mRNAs across breast cancer subtypes using qRT-PCR. HER2, human epidermal growth factor receptor 2; TNBC, triple-negative breast cancer; *CXCL8*, C-X-C Motif Chemokine Ligand 8 (also known as Interleukin-8, IL-8); *BCL2*, B-Cell Lymphoma 2, *BAX*, BCL2-Associated X Protein; *CASP1,* Caspase-1; *CASP9*, Caspase-9; *TP53*, Tumor Protein p53; *CDKN1A*, Cyclin-Dependent Kinase Inhibitor 1A (p21); *CDKN1B*, Cyclin-Dependent Kinase Inhibitor 1B (p27); *MMP9*, Matrix Metallopeptidase 9.

qRT-PCR analysis confirmed the differential expression patterns identified in the microarray experiment. *CXCL8, BAX*, *CASP1*, *CASP9*, *TP53*, and *MMP9* were progressively upregulated across all breast cancer subtypes, with the strongest increases observed in non-luminal HER2+ and TNBC tumors. *BCL2* showed marked overexpression in luminal subtypes but was downregulated in HER2-enriched and TNBC samples. In contrast, *CDKN1A* and *CDKN1B* exhibited subtype-specific suppression, most prominently in TNBC, consistent with loss of cell-cycle control in aggressive tumors. Overall, qRT-PCR results closely mirrored the microarray findings, confirming subtype-dependent molecular alterations (Figure 2).

### 3.4 Prediction of pyroptosis-related gene expression regulation by miRNAs

Differential expression of miRNAs was determined directly from microarray data by comparing tumor and control tissues across breast cancer subtypes. Subsequently, putative miRNA–mRNA regulatory interactions were identified using *in silico* target prediction algorithms, and only high-confidence pairs with target scores between 89 and 97 were selected for further analysis (Table 3). Importantly, the predicted interactions were evaluated independently of expression directionality, as miRNA-mediated regulation does not necessarily result in a linear inverse relationship at the transcript level.

**TABLE 3:** The expression profile of miRNAs potentially regulated by selected mRNAs in different breast cancer subtype tissues in comparison to control tissues.

mRNA	miRNA	Target score	Luminal A vs. Control (FC)	Luminal B HER2− vs. control (FC)	Luminal B HER2+ vs. control (FC)	Non-luminal HER2+ vs. control (FC)	TNBC vs. control (FC)
*CXCL8*	hsa-miR-140-3p	94	3.08 ± 0.45*	3.08 ± 0.21*	3.33 ± 0.31*	5.76 ± 0.19*	7.65 ± 0.19*
*BAX*	hsa-miR-1843	90	3.18 ± 0.19*	3.18 ± 0.15*	4.45 ± 0.43*	5.98 ± 0.21*	9.12 ± 0.22*
*CASP9*	hsa-miR-124-3p	91	3.10 ± 0.23*	3.10 ± 0.19*	3.91 ± 0.12*	4.15 ± 0.19*	4.76 ± 0.29*
*TP53*	hsa-miR-300	89	3.11 ± 0.19*	3.11 ± 0.42*	3.41 ± 0.21*	5.91 ± 0.32*	6.19 ± 0.31*
*CDKN1A*	hsa-miR-608	95	3.45 ± 0.23*	3.45 ± 0.23*	4.21 ± 0.19*	4.98 ± 0.21*	−4.18 ± 0.19*
*CDKN1B*	hsa-miR-30d-3p	97	3.11 ± 0.19*	3.19 ± 0.18*	3.89 ± 0.32*	−3.14 ± 0.43*	−6.19 ± 0.76*
	hsa-miR-30a-3p	97	3.11 ± 0.18*	3.17 ± 0.18*	3.99 ± 0.21*	−3.14 ± 0.31*	−6.19 ± 0.54*

HER2, human epidermal growth factor receptor 2; TNBC, triple-negative breast cancer; *CXCL8*, C-X-C Motif Chemokine Ligand 8 (also known as Interleukin-8, IL-8); *BAX*, BCL2-Associated X Protein; *CASP9*, Caspase-9; *TP53*, Tumor Protein p53; *CDKN1A*, Cyclin-Dependent Kinase Inhibitor 1A (p21); *CDKN1B*, Cyclin-Dependent Kinase Inhibitor 1B (p27); *, statistically silniance differences in comparison to a control (p < 0.05).

Prediction analysis identified several high-confidence miRNA–mRNA pairs corresponding to genes that were consistently differentially expressed across breast cancer subtypes. *CXCL8*, predicted to be regulated by hsa-miR-140-3p, was markedly upregulated in all molecular subtypes, with the highest expression observed in TNBC (7.65 ± 0.19). Similarly, *BAX* and *CASP9*, paired with hsa-miR-1843 and hsa-miR-124-3p, respectively, showed progressive upregulation, reaching maximal expression levels in aggressive tumor subtypes. *TP53*, linked to hsa-miR-300, exhibited a comparable subtype-dependent increase.

In contrast, *CDKN1A* and *CDKN1B*, predicted targets of hsa-miR-608 and hsa-miR-30 family members (hsa-miR-30d-3p and hsa-miR-30a-3p), demonstrated divergent expression patterns. While both genes remained upregulated in luminal subtypes, they were markedly downregulated in non-luminal HER2-positive and TNBC tumors, with *CDKN1B* showing particularly strong suppression in TNBC (−6.19 ± 0.76). These findings indicate that predicted miRNA–mRNA interactions should be interpreted as biologically plausible regulatory relationships rather than direct expression correlations.

### 3.5 ELISA-based quantification of pyroptosis-related proteins across breast cancer subtypes

Table 4 summarizes the estimated concentrations of nine pyroptosis-related proteins measured by ELISA in control tissue and across five breast cancer subtypes. All proteins showed significant differences compared with controls (*p* < 0.05). CXCL8, BAX, CASP1, CASP9, TP53, and MMP9 displayed a progressive increase from luminal tumors to the most aggressive subtypes, reaching the highest concentrations in non-luminal HER2+ and TNBC. In contrast, BCL2, CDKN1A (p21), and CDKN1B (p27) were elevated in luminal cancers but markedly reduced in non-luminal HER2+ and TNBC, reflecting subtype-specific differences in apoptotic and cell-cycle regulation.

**TABLE 4:** Estimated concentrations (mean ± SD) of pyroptosis-associated proteins in control and breast cancer tissues measured by ELISA.

Protein	Control tissue	Luminal A	Luminal B HER2–	Luminal B HER2+	Non-luminal HER2+	TNBC
CXCL8 (pg/mL)	82.4 ± 9.8	152.7 ± 14.2*	184.9 ± 16.1*	362.5 ± 22.4*	918.3 ± 41.7*	1248.6 ± 55.2*
BCL2 (ng/mL)	5.3 ± 0.6	24.8 ± 2.1*	17.6 ± 1.9*	20.9 ± 1.4*	3.2 ± 0.4*	2.1 ± 0.3*
BAX (ng/mL)	2.2 ± 0.3	5.4 ± 0.5*	9.3 ± 0.9*	15.7 ± 1.2*	34.4 ± 2.7*	46.8 ± 3.8*
CASP1 (ng/mL)	0.28 ± 0.04	1.19 ± 0.09*	2.11 ± 0.18*	3.62 ± 0.24*	6.73 ± 0.41*	10.84 ± 0.69*
CASP9 (ng/mL)	1.47 ± 0.17	4.12 ± 0.38*	6.48 ± 0.53*	8.23 ± 0.49*	15.29 ± 0.91*	20.88 ± 1.34*
P53 (pg/mL)	118.3 ± 10.2	298.4 ± 21.6*	352.9 ± 24.1*	701.8 ± 33.5*	1134.2 ± 52.6*	1622.4 ± 68.3*
CDKN1A/p21 (ng/mL)	1.18 ± 0.12	7.83 ± 0.71*	10.21 ± 0.89*	11.92 ± 0.97*	3.14 ± 0.29*	1.07 ± 0.11*
CDKN1B/p27 (ng/mL)	1.36 ± 0.15	6.14 ± 0.53*	8.31 ± 0.72*	2.18 ± 0.19*	1.04 ± 0.10*	0.79 ± 0.08*
MMP9 (pg/mL)	310.4 ± 34.1	628.9 ± 51.3*	812.7 ± 62.8*	1432.5 ± 98.6*	5128.7 ± 243.9*	8234.6 ± 387.5*

HER2, human epidermal growth factor receptor 2; TNBC, triple-negative breast cancer; *CXCL8*, C-X-C Motif Chemokine Ligand 8 (also known as Interleukin-8, IL-8); *CXCL8*, C-X-C Motif Chemokine Ligand 8 (also known as Interleukin-8, IL-8); *BCL2*, B-Cell Lymphoma 2, *BAX*, BCL2-Associated X Protein; *CASP1,* Caspase-1; *CASP9*, Caspase-9; *TP53*, Tumor Protein p53; *CDKN1A*, Cyclin-Dependent Kinase Inhibitor 1A (p21); *CDKN1B*, Cyclin-Dependent Kinase Inhibitor 1B (p27); *MMP9*, Matrix Metallopeptidase 9; *, statistically silniance differences in comparison to a control (p < 0.05).

### 3.6 Temporal dynamics of mRNA and protein expression profiles in women with fibroadenoma

The transcriptional response showed a distinct early activation pattern. *CXCL8*, *BAX*, *CASP1*, *CASP9*, and *MMP9* demonstrated a rapid rise already at T1, reaching peak expression at T2 (8–12 h), followed by a gradual decline between T3–T6. *TP53* increased moderately, with a significant elevation at T3. *BCL2* showed only minimal and transient upregulation at T2–T3, while *CDKN1A* and *CDKN1B* exhibited mild, non-significant fluctuations without pronounced temporal changes. Overall, the data indicate an early inflammatory and apoptotic transcriptional peak with subsequent normalization within 1–3 months (Table 5).

**TABLE 5:** Longitudinal changes in mRNA expression of selected genes in women with fibroadenoma.

mRNA	T1 vs. T0	T2 vs. T0	T3 vs. T0	T4 vs. T0	T5 vs. T0	T6 vs. T0
*CXCL8*	1.45 ± 0.18*	2.10 ± 0.25*	1.90 ± 0.22*	1.35 ± 0.16*	1.10 ± 0.12	1.02 ± 0.10
*BCL2*	1.05 ± 0.09	1.10 ± 0.11*	1.08 ± 0.10*	1.03 ± 0.09	1.00 ± 0.08	0.98 ± 0.08
*BAX*	1.30 ± 0.14	1.80 ± 0.20*	1.75 ± 0.19*	1.30 ± 0.15*	1.10 ± 0.11	1.00 ± 0.10
*CASP1*	1.35 ± 0.15	1.95 ± 0.21*	1.85 ± 0.20*	1.40 ± 0.16*	1.12 ± 0.12	1.03 ± 0.10
*CASP9*	1.25 ± 0.13	1.70 ± 0.18*	1.65 ± 0.17*	1.28 ± 0.14	1.08 ± 0.11	1.01 ± 0.09
*TP53*	1.20 ± 0.12	1.55 ± 0.17	1.50 ± 0.16*	1.25 ± 0.13	1.05 ± 0.10	1.00 ± 0.09
*CDKN1A*	1.15 ± 0.12	1.35 ± 0.15	1.30 ± 0.14	1.10 ± 0.12	1.02 ± 0.10	0.98 ± 0.09
*CDKN1B*	1.10 ± 0.11	1.25 ± 0.13	1.22 ± 0.13	1.08 ± 0.11	1.00 ± 0.09	0.98 ± 0.08
*MMP9*	1.40 ± 0.16*	2.00 ± 0.23*	1.85 ± 0.21*	1.45 ± 0.17*	1.15 ± 0.13	1.05 ± 0.11

*CXCL8*, C-X-C Motif Chemokine Ligand 8 (also known as Interleukin-8, IL-8); *BCL2*, B-Cell Lymphoma 2, *BAX*, BCL2-Associated X Protein; *CASP1,* Caspase-1; *CASP9*, Caspase-9; *TP53*, Tumor Protein p53; *CDKN1A*, Cyclin-Dependent Kinase Inhibitor 1A (p21); *CDKN1B*, Cyclin-Dependent Kinase Inhibitor 1B (p27); *MMP9*, Matrix Metallopeptidase 9; *, statistically significant differences in comparison to a control (p < 0.05).

Protein levels paralleled the mRNA dynamics, with the highest concentrations for CXCL8, BAX, CASP1, CASP9, P53, CDKN1A, CDKN1B, and MMP9 observed at T2, and in several cases also at T1 and T3. A progressive reduction was noted thereafter, with values approaching baseline by T5–T6. BCL2 protein remained relatively stable, exhibiting only minor elevations at early time points (Table 6).

**TABLE 6:** Temporal profile of protein concentrations corresponding to selected genes in women with fibroadenoma.

Protein	T0 (baseline)	T1 (30–60 min)	T2 (8–12 h)	T3 (48–72 h)	T4 (7 days)	T5 (1 month)	T6 (3 months)
CXCL8 (pg/mL)	65.2 ± 8.1	92.4 ± 10.5*	138.7 ± 14.9*	125.3 ± 13.8*	88.6 ± 9.7	70.9 ± 8.4*	66.3 ± 7.9
BCL2 (ng/mL)	2.8 ± 0.3	3.0 ± 0.3	3.2 ± 0.4*	3.1 ± 0.3*	2.9 ± 0.3*	2.8 ± 0.3*	2.7 ± 0.3*
BAX (ng/mL)	1.4 ± 0.2	1.9 ± 0.2*	2.6 ± 0.3*	2.5 ± 0.3*	1.9 ± 0.2	1.6 ± 0.2*	1.4 ± 0.2*
CASP1 (ng/mL)	0.18 ± 0.03	0.32 ± 0.04*	0.52 ± 0.06*	0.49 ± 0.05*	0.30 ± 0.04*	0.22 ± 0.03*	0.19 ± 0.03*
CASP9 (ng/mL)	1.10 ± 0.14	1.55 ± 0.18*	2.20 ± 0.25*	2.05 ± 0.23*	1.50 ± 0.18*	1.25 ± 0.15*	1.12 ± 0.14*
P53 (pg/mL)	95.3 ± 9.6	130.8 ± 12.7*	178.4 ± 16.9*	169.2 ± 15.8*	122.7 ± 11.3*	102.9 ± 10.1*	96.5 ± 9.4*
CDKN1A (ng/mL)	0.95 ± 0.10	1.20 ± 0.13*	1.40 ± 0.15*	1.35 ± 0.14*	1.05 ± 0.11*	0.97 ± 0.10*	0.94 ± 0.09*
CDKN1B (ng/mL)	1.05 ± 0.11	1.25 ± 0.13*	1.45 ± 0.16*	1.40 ± 0.15*	1.15 ± 0.12**	1.03 ± 0.11*	1.00 ± 0.10*
MMP9 (pg/mL)	260.5 ± 28.7	340.9 ± 35.1*	520.6 ± 49.8*	485.3 ± 45.7*	330.1 ± 32.2*	280.9 ± 29.4*	265.7 ± 27.9*

*CXCL8*, C-X-C Motif Chemokine Ligand 8 (also known as Interleukin-8, IL-8); *BCL2*, B-Cell Lymphoma 2, *BAX*, BCL2-Associated X Protein; *CASP1,* Caspase-1; *CASP9*, Caspase-9; *TP53*, Tumor Protein p53; *CDKN1A*, Cyclin-Dependent Kinase Inhibitor 1A (p21); *CDKN1B*, Cyclin-Dependent Kinase Inhibitor 1B (p27); *MMP9*, Matrix Metallopeptidase 9; *, statistically significant differences in comparison to a control (p < 0.05).

### 3.7 Protein–protein interaction (PPI) network analysis of pyroptosis-associated proteins

**FIGURE 3:**
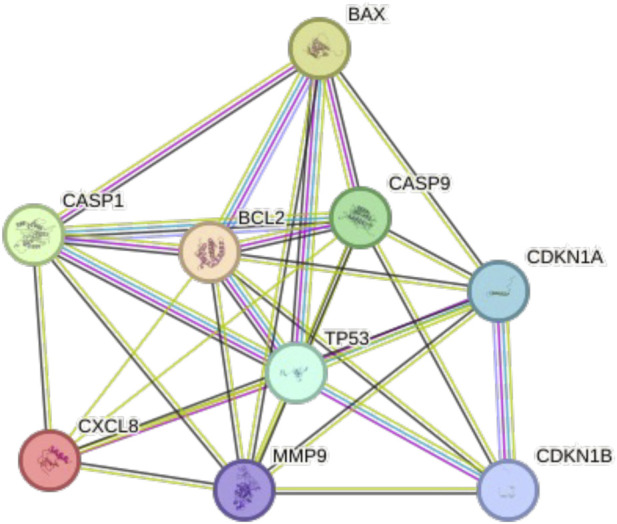
STRING-derived protein–protein interaction network of nine differentially expressed pyroptosis-associated genes shared across all breast cancer subtypes.

The STRING-based PPI analysis performed on the nine subtype-independent, differentially expressed mRNAs revealed a highly interconnected molecular network (Figure 3). The network consisted of 9 nodes representing the encoded proteins and 31 edges, substantially exceeding the expected 10 interactions for a network of this size. The average node degree was 6.89, indicating that most proteins were linked to nearly seven interaction partners, reflecting strong functional connectivity. The average local clustering coefficient of 0.883 further supported the presence of densely interconnected clusters within the network. Importantly, the PPI enrichment p-value (6.31 × 10^−8^) demonstrated that the observed interactions were significantly more frequent than would occur by chance, confirming that these proteins are functionally related and likely engage in shared biological processes. The central positioning of TP53, BCL2, BAX, CASP1, and CASP9 highlights their coordinated involvement in regulated cell death pathways, including apoptosis–pyroptosis crosstalk (Figure 3).

### 3.8 PI across breast cancer subtypes

The PI demonstrated a clear and progressive increase from luminal to more aggressive breast cancer subtypes, indicating a gradual shift toward enhanced inflammatory and pyroptosis-associated cell-death activity (Table 7). Luminal A tumors exhibited the lowest PI value (−0.67), consistent with minimal activation of pyroptotic signaling pathways. Luminal B HER2– tumors showed a near-neutral PI (0.13), whereas luminal B HER2+ tumors displayed a moderate increase (PI = 3.33). In contrast, markedly elevated PI values were observed in aggressive subtypes, with non-luminal HER2-positive tumors reaching a PI of 11.65 and TNBC exhibiting the highest PI (18.46), reflecting pronounced activation of pyroptosis-related molecular programs.

**TABLE 7:** Pyroptosis Index calculated from nine core pyroptosis-associated genes.

Breast cancer subtype	Pyroptosis index (PI)
Luminal A	−0.67
Luminal B HER2–	0.13
Luminal B HER2+	3.33
Non-luminal HER2+	11.65
TNBC	18.46

HER2, human epidermal growth factor receptor 2; TNBC, triple-negative breast cancer.

### 3.9 IAS across breast cancer subtypes

The IAS values followed a pattern closely paralleling that of the PI, with progressively increasing values across breast cancer subtypes of rising biological aggressiveness (Table 8). Luminal A tumors showed the lowest IAS (3.35), indicative of relatively modest inflammasome activity. Incremental increases were observed in luminal B HER2– (IAS = 3.92) and luminal B HER2+ tumors (IAS = 5.15). Substantially higher IAS values were detected in non-luminal HER2-positive tumors (7.00) and TNBC (8.41), reflecting intensified activation of inflammasome-associated signaling pathways.

**TABLE 8:** Inflammasome Activation Score (IAS) based on 10 inflammasome-related genes.

Breast cancer subtype	IAS
Luminal A	3.35
Luminal B HER2–	3.92
Luminal B HER2+	5.15
Non-luminal HER2+	7.00
TNBC	8.41

HER2, human epidermal growth factor receptor 2; TNBC, triple-negative breast cancer.

This gradient corresponded with the stepwise upregulation of key inflammasome-related genes, including IL1B, IL18, NLRP3, CASP1, and additional inflammatory regulators. Detailed log_2_ fold-change values for individual inflammasome-associated transcripts across molecular subtypes are provided in Supplementary Table S1.

### 3.10 Overall survival (OS) analysis

**FIGURE 4:**
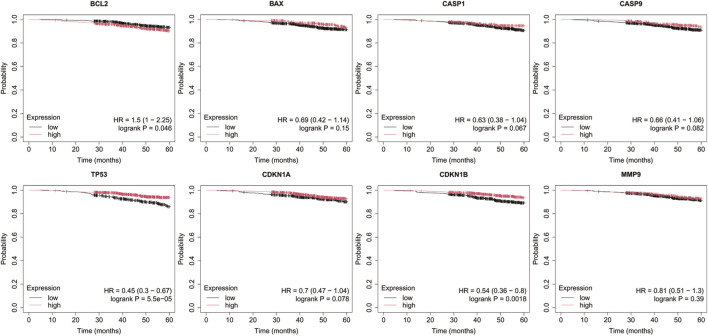
Overall survival analysis in luminal A cancer. *BCL2*, B-Cell Lymphoma 2, *BAX*, BCL2-Associated X Protein; *CASP1*, Caspase-1; *CASP9*, Caspase-9; *TP53*, Tumor Protein p53; *CDKN1A*, Cyclin-Dependent Kinase Inhibitor 1A (p21); *CDKN1B*, Cyclin-Dependent Kinase Inhibitor 1B (p27); *MMP9*, Matrix Metallopeptidase 9.

**FIGURE 5:**
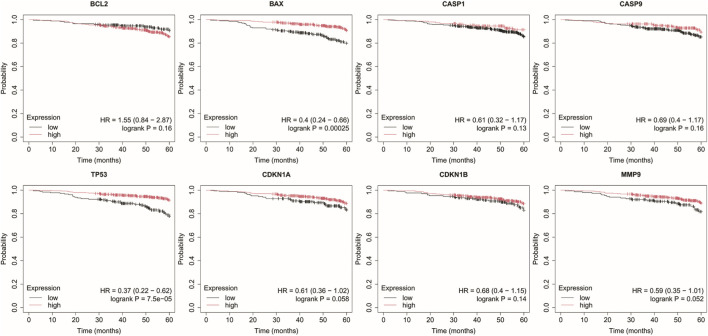
Overall survival analysis in HER2– luminal B cancer. *BCL2*, B-Cell Lymphoma 2, *BAX*, BCL2-Associated X Protein; *CASP1*, Caspase-1; *CASP9*, Caspase-9; *TP53*, Tumor Protein p53; *CDKN1A*, Cyclin-Dependent Kinase Inhibitor 1A (p21); *CDKN1B*, Cyclin-Dependent Kinase Inhibitor 1B (p27); *MMP9*, Matrix Metallopeptidase 9.

**FIGURE 6:**
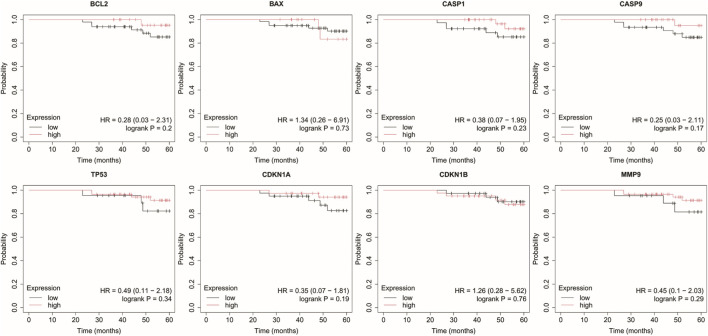
Overall survival analysis in HER2+ luminal B cancer. *BCL2*, B-Cell Lymphoma 2, *BAX*, BCL2-Associated X Protein; *CASP1*, Caspase-1; *CASP9*, Caspase-9; *TP53*, Tumor Protein p53; *CDKN1A*, Cyclin-Dependent Kinase Inhibitor 1A (p21); *CDKN1B*, Cyclin-Dependent Kinase Inhibitor 1B (p27); *MMP9*, Matrix Metallopeptidase 9.

**FIGURE 7:**
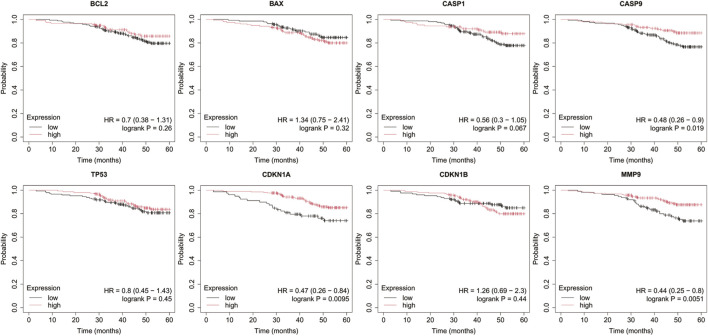
Overall survival analysis in non-luminal HER2+ cancer. *BCL2*, B-Cell Lymphoma 2, *BAX*, BCL2-Associated X Protein; *CASP1*, Caspase-1; *CASP9*, Caspase-9; *TP53*, Tumor Protein p53; *CDKN1A*, Cyclin-Dependent Kinase Inhibitor 1A (p21); *CDKN1B*, Cyclin-Dependent Kinase Inhibitor 1B (p27); *MMP9*, Matrix Metallopeptidase 9.

**FIGURE 8:**
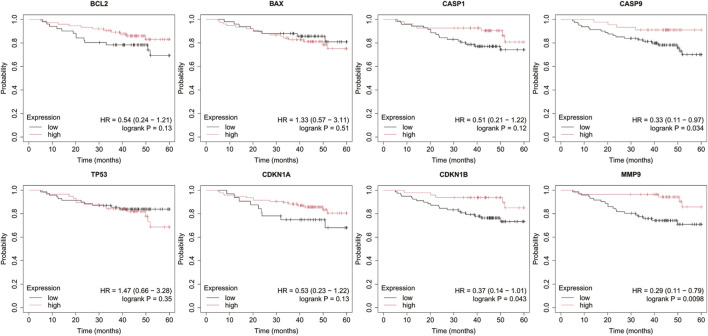
Overall survival analysis in TNBC. *BCL2*, B-Cell Lymphoma 2, *BAX*, BCL2-Associated X Protein; *CASP1*, Caspase-1; *CASP9*, Caspase-9; *TP53*, Tumor Protein p53; *CDKN1A*, Cyclin-Dependent Kinase Inhibitor 1A (p21); *CDKN1B*, Cyclin-Dependent Kinase Inhibitor 1B (p27); *MMP9*, Matrix Metallopeptidase 9.

Analysis was performed for *BCL2*, *BAX*, *CASP1*, *CASP9*, *TP53*, *CDKN1A*, *CDKN1B*, and *MMP9* with a follow-up threshold of 60 months. The database did not contain data for the *CXCL8* gene, therefore it was omitted from this analysis (Figures 4–8).

In luminal A subtype, high *BCL2* expression with low *TP53* and *CDKN1B* expression were associated with worse OS (Figure 5).

Low *BAX* and *TP53* expression were associated with poorer OS in HER2– luminal B cancer.

In the HER2+ luminal B subtype, the change in expression of the studied genes is probably not associated with worse OS.

In turn, in non-luminal HER2+ cancer, low levels of *CDKN1A* and *MMP9* promote worse OS.

In TNBC, low levels of *CASP9*, *CDKN1B*, and *MMP9* were associated with poorer OS.

## 4 Discussion

This study provides a comprehensive, multi-level characterization of pyroptosis-associated alterations in breast cancer, integrating mRNA and protein expression, predicted miRNA–mRNA interactions, and temporal transcriptional changes observed in a fibroadenoma cryoablation model. Pyroptosis is increasingly recognized as a context-dependent process in cancer, exerting both tumor-suppressive and tumor-promoting effects depending on the duration, intensity, and cellular context of inflammasome activation (Chen et al., 2024; Gao et al., 2022; Wu J. et al., 2022). In the present study, we intentionally focused on genes involved in the initiation and activation stages of pyroptosis, including inflammatory mediators, caspases, cell-cycle regulators, and their microRNA modulators. This strategy was chosen to capture early and regulatory molecular events that shape pyroptotic signaling in breast cancer. Genes primarily responsible for the execution phase of pyroptosis, such as gasdermin family members (e.g., GSDMD), were therefore not the main subject of analysis and are discussed only in a contextual manner. Across five molecular subtypes, we identified a core set of nine genes—*CXCL8*, *BCL2*, *BAX*, *CASP1*, *CASP9*, *TP53*, *CDKN1A*, *CDKN1B*, and *MMP9*—that robustly differentiate tumor tissue from matched controls. Together, these coordinately dysregulated transcripts form a pyroptosis-related molecular signature that reveals how inflammatory cell death interfaces with apoptosis, cell-cycle regulation, and extracellular matrix remodeling in both indolent and aggressive breast cancer phenotypes. The accompanying miRNA analyses further highlight an additional layer of regulation that shapes the balance between inflammatory tumor promotion and programmed cell death.

Among the identified markers, CXCL8 (IL-8) emerged as the most strongly and consistently upregulated gene across all breast cancer subtypes, with particularly high expression in HER2-enriched and TNBC tumors. This pattern supports CXCL8 as a central mediator of breast cancer–associated inflammation (Stępień et al., 2024). As a downstream effector of inflammasome activation, CXCL8 amplifies NF-κB signaling, promotes angiogenesis, and facilitates recruitment of immunosuppressive myeloid cells (Stępień et al., 2024; Tang et al., 2025). The predicted regulatory miR-140-3p, a known tumor suppressor (Huang et al., 2019), appeared insufficient to counterbalance CXCL8 overexpression in high-grade tumors, likely contributing to the sustained inflammatory microenvironment characteristic of aggressive disease (Rosado-Sanz et al., 2025).

A contrasting, subtype-dependent pattern was observed for the apoptotic regulators BCL2 and BAX. Luminal tumors showed elevated BCL2 expression, consistent with preserved survival signaling and partial apoptotic control (Vogler et al., 2025), and high BCL2 levels were associated with worse overall survival in luminal A patients. In contrast, HER2+ and TNBC tumors exhibited marked BCL2 suppression accompanied by progressive BAX upregulation. This imbalance suggests a shift toward mitochondrial permeabilization and inflammatory caspase activation in aggressive tumors (Samia et al., 2024; Li et al., 2022). Reduced expression of the predicted BAX regulator miR-1843 may further potentiate this effect (Li et al., 2022). Together with increased CASP1 and CASP9 levels, these findings indicate a hybrid cell-death phenotype integrating apoptotic and pyroptotic mechanisms (Li et al., 2022; Scientia, 2025), supported by evidence that BAX/BAK pores can initiate gasdermin-mediated pyroptosis (Slaufova et al., 2023).

Consistent upregulation of CASP1 further underscores the relevance of inflammasome signaling in breast cancer (Chen et al., 2023; Wu L. et al., 2022), particularly in HER2-enriched and TNBC tumors characterized by a pro-inflammatory microenvironment. Elevated CASP9 expression highlights extensive crosstalk between intrinsic apoptosis and pyroptosis (Liu X. et al., 2024; Wang et al., 2023), potentially facilitated by downregulation of its regulatory miRNA miR-124-3p (Liu Y. et al., 2024). Such coordinated caspase activation may sustain chronic inflammatory signaling and promote tumor progression through mixed cell-death dynamics rather than reliance on a single pathway (Li et al., 2022; Liu X. et al., 2024).

TP53 expression was markedly increased in aggressive subtypes, linking genomic instability with inflammatory cell-death regulation (Hwang et al., 2024). Elevated p53 protein levels may reflect stress responses or accumulation of mutant isoforms with prolonged stability (Wang et al., 2025). In contrast, lower TP53 expression in luminal A and HER2–luminal B tumors was associated with poorer survival outcomes. The predicted regulatory miR-300, implicated in cell-cycle control and EMT, may modulate TP53 activity (Wang and Yu, 2016). Given p53’s central role in regulating BAX, caspases, and gasdermins, its expression positions it at the intersection of apoptotic and pyroptotic pathways (Wang et al., 2025; Wang et al., 2021).

Cell-cycle inhibitors CDKN1A (p21) and CDKN1B (p27) displayed a clear dichotomy between luminal and aggressive tumors. Their upregulation in luminal cancers reflects partial cell-cycle restraint, whereas profound suppression in HER2+ and TNBC tumors indicates loss of G1/S control and enhanced proliferative capacity. Downregulation of their regulatory miRNAs (miR-608, miR-30a-3p, and miR-30d-3p) likely contributes to this phenotype (Wiśniewski et al., 2018). Reduced p21/p27 expression may also increase susceptibility to caspase activation, facilitating shifts between apoptotic and pyroptotic cell death (Akhter et al., 2021). This shift underscores how loss of cell-cycle control and heightened inflammatory signaling co-evolve in aggressive breast cancers (Wang et al., 2025). Consistently, low CDKN1A and CDKN1B levels were associated with worse prognosis in non-luminal HER2+ cancer and TNBC, respectively.

Upregulation of MMP9, particularly in HER2+ and TNBC tumors, adds an extracellular dimension to the pyroptosis-associated signature (Augoff et al., 2022). As a key matrix-degrading enzyme, MMP9 promotes invasion and metastasis through extracellular matrix remodeling (Lee et al., 2024). Its induction by inflammasome-associated cytokines, including CXCL8 and IL-1β, suggests that elevated MMP9 reflects downstream consequences of tumor-associated pyroptosis. The coordinated increase in CXCL8, CASP1, and MMP9 highlights an inflammatory–matrix remodeling axis that may drive metastatic potential in aggressive subtypes (Chen et al., 2023).

Integrating these findings with the predicted miRNA–mRNA interactions reveals a unifying regulatory pattern in aggressive breast tumors. Notably, the miRNA–mRNA relationships presented here are based on target prediction and expression context rather than correlation analysis, as miRNA-mediated regulation does not require a strict inverse relationship at the mRNA level. Several tumor-suppressive miRNAs—miR-140-3p, miR-124-3p, miR-300, miR-30a-3p, miR-30d-3p, and miR-608—were insufficiently expressed to restrain inflammatory, apoptotic, and proliferative signaling. Loss of miRNA-mediated control likely amplifies pyroptosis-associated pathways, potentiates caspase-driven cell death, and facilitates unchecked cell-cycle progression (Lee et al., 2025; Liu Y. et al., 2024). These findings emphasize miRNAs as central integrators of inflammatory signaling and cell-death plasticity in high-grade breast cancer (Lee et al., 2025; Liu Y.et al., 2024; Stămat et al., 2023).

Consistent with these observations, both the PI and IAS demonstrated a clear gradient across breast cancer subtypes, with minimal activation in luminal tumors and progressively higher values in HER2-enriched and TNBC cancers. This pattern suggests reliance of aggressive tumors on sustained IL-1β/IL-18 signaling, caspase-1 activation, and downstream matrix remodeling to support tumor progression and immune evasion (Chen et al., 2023). The concordance between PI and IAS reinforces the interconnected nature of pyroptosis and inflammasome biology and aligns with established transcriptomic signatures of aggressive breast cancer (Wang et al., 2023; Zhang et al., 2023).

The fibroadenoma cohort provided insight into the systemic inflammatory response associated with acute, non-malignant tissue injury rather than direct tissue-level pyroptosis. Because RNA was extracted from peripheral blood, the observed transcriptional changes primarily reflect activation dynamics of circulating immune cells responding to cryoablation-induced local inflammation. Cryoablation is known to induce rapid local tissue damage accompanied by immune cell recruitment and cytokine release, which can be captured indirectly through changes in blood immune cell transcriptomes, even though this does not constitute a direct measure of pyroptosis activation within the affected tissue itself. Cryoablation induced rapid but transient increases in CXCL8, BAX, CASP1, CASP9, and MMP9, followed by normalization within one to 3 months, reflecting resolution of acute inflammation and tissue repair (Wang et al., 2023). In contrast, breast cancer tissue exhibited a consistent and subtype-dependent elevation of pyroptosis- and inflammasome-associated markers at the time of surgical resection, indicating a fundamentally different inflammatory context from the transient systemic response observed following benign tissue injury (Zheng et al., 2024). This interpretation is supported by evidence that tumor microenvironments can continuously provide inflammatory stimuli—such as IL-1β/IL-18 signaling, NF-κB activation, and immune–tumor cell interactions—that sustain inflammasome-related pathways in aggressive breast cancer.

Clinically, elevated expression of CXCL8, CASP1, CASP9, MMP9, and BAX—particularly in HER2-enriched and TNBC tumors—may serve as biomarkers of aggressiveness and aid in risk stratification. Therapeutically, targeting the CXCL8/CXCR1/2 axis or inhibiting caspase-1 with agents such as VX-765 or belnacasan may attenuate inflammasome-driven tumor progression (Zheng et al., 2024).

Several limitations should be acknowledged. Microarray-based analyses lack the resolution of RNA sequencing, predicted miRNA–mRNA interactions require functional validation, and ELISA does not distinguish active from inactive caspase forms. The fibroadenoma model cannot fully recapitulate malignant cell-death mechanisms, and absence of gasdermin and mutational profiling (e.g., TP53, BRCA1/2) limits mechanistic resolution. Future studies employing functional genomics, single-cell approaches, and spatial proteomics are warranted. Importantly, longitudinal persistence of pyroptosis activation was not directly assessed in the breast cancer cohort; therefore, ‘sustained’ activation refers to consistent cross-sectional elevation at resection rather than temporal dynamics within individual patients.

In summary, this study identifies a conserved pyroptosis-associated molecular signature distinguishing breast cancer from normal tissue across all major molecular subtypes. Coordinated dysregulation of CXCL8, BAX, CASP1, CASP9, TP53, and MMP9, together with subtype-specific suppression of BCL2, CDKN1A, and CDKN1B, reveals an integrated inflammatory–apoptotic network that intensifies with tumor aggressiveness. miRNA dysregulation further amplifies these processes, positioning pyroptosis as a multifaceted driver of breast cancer biology with diagnostic, prognostic, and therapeutic relevance.

## Data Availability

The original contributions presented in the study are publicly available. This data can be found here: panfil, agata (2026). Integrated mRNA and Protein Expression Database of Pyroptosis Markers in Breast Cancer. figshare. Dataset. 10.6084/m9.figshare.31331875.v1
