# Supplementary material for: Integrated transcriptomic, protein, and MicroRNA profiling reveals a conserved pyroptosis-related molecular signature across breast cancer subtypes
Source: Front Mol Biosci. 2026 Mar 4;13:1759944. doi: 10.3389/fmolb.2026.1759944 (PMC12995627; doi:10.3389/fmolb.2026.1759944)
Supplement: Supplementary file 1 [file Table1.docx]

Supplementary Table S1. log₂ fold-change (mean ± SD) of inflammasome-related genes across breast cancer subtypes

| mRNA | Log_2_FC | | | | |
| --- | --- | --- | --- | --- | --- |
|  | Luminal A vs. C | Luminal B HER2– vs. C | Luminal B HER2+ vs. C | Non-luminal HER2+ vs. C | TNBC vs. C |
| *IL1B* | 3.29 ± 0.28* | 3.50 ± 0.31* | 5.01 ± 0.47* | 7.00 ± 0.63* | 8.51± 0.79* |
| *IL18* | 3.11 ± 0.26* | 3.42 ± 0.33* | 4.82 ± 0.44* | 6.81 ± 0.62* | 8.23 ± 0.74* |
| *NLRP3* | 3.01 ± 0.27* | 3.25 ± 0.29* | 4.54 ± 0.41* | 7.21 ± 0.67* | 8.92 ± 0.85* |
| *PYCARD (ASC)* | 3.25 ± 0.30* | 3.61 ± 0.34* | 5.32 ± 0.49* | 7.45 ± 0.69* | 9.11 ± 0.88* |
| *TLR9* | 3.70 ± 0.33* | 4.43 ± 0.38** | 5.63 ± 0.50* | 6.22 ± 0.56* | 7.85 ± 0.71* |
| *RIPK1* | 3.54 ± 0.32* | 4.12 ± 0.37* | 5.23 ± 0.48* | 7.58 ± 0.72* | 9.14 ± 0.86* |
| *TNF* | 3.32 ± 0.29* | 3.84 ± 0.35* | 5.55 ± 0.51** | 7.11 ± 0.66* | 9.20 ± 0.87* |
| *STING1* | 3.19 ± 0.30* | 3.92 ± 0.36* | 5.64 ± 0.52* | 7.32 ± 0.68* | 8.91 ± 0.84* |
| *JAK3* | 3.43 ± 0.31* | 4.07 ± 0.37* | 5.72 ± 0.53* | 7.62 ± 0.73* | 9.33 ± 0.91* |
